# Metalloregulation of *Helicobacter pylori* physiology and pathogenesis

**DOI:** 10.3389/fmicb.2015.00911

**Published:** 2015-09-02

**Authors:** Kathryn P. Haley, Jennifer A. Gaddy

**Affiliations:** ^1^Tennessee Valley Healthcare Services, Department of Veterans AffairsNashville, TN, USA; ^2^Department of Medicine, Vanderbilt University School of MedicineNashville, TN, USA

**Keywords:** *Helicobacter pylori*, gene, regulation, metal, virulence

## Abstract

*Helicobacter pylori* is a Gram-negative spiral-shaped bacterium that colonizes over half of the world's population. Chronic *H. pylori* infection is associated with increased risk for numerous disease outcomes including gastritis, dysplasia, neoplasia, B-cell lymphoma of mucosal-associated lymphoid tissue (MALT lymphoma), and invasive adenocarcinoma. The complex interactions that occur between pathogen and host are dynamic and exquisitely regulated, and the relationship between *H. pylori* and its human host are no exception. To successfully colonize, and subsequently persist, within the human stomach *H. pylori* must temporally regulate numerous genes to ensure localization to the gastric lumen and coordinated expression of virulence factors to subvert the host's innate and adaptive immune response. *H. pylori* achieves this precise gene regulation by sensing subtle environmental changes including host-mediated alterations in nutrient availability and responding with dramatic global changes in gene expression. Recent studies revealed that the presence or absence of numerous metal ions encountered in the lumen of the stomach, or within host tissues, including nickel, iron, copper and zinc, can influence regulatory networks to alter gene expression in *H. pylori*. These expression changes modulate the deployment of bacterial virulence factors that can ultimately influence disease outcome. In this review we will discuss the environmental stimuli that are detected by *H. pylori* as well as the *trans* regulatory elements, specifically the transcription regulators and transcription factors, that allow for these significant transcriptional shifts.

## Introduction

*Helicobacter pylori* colonizes over half of the world's population making it arguably the most successful bacterial pathogen (Atherton and Blaser, 2009). *H. pylori* is uniquely adapted to colonize the human stomach and is the dominant microorganism within the gastric niche (Bik et al., 2006; Cover and Blaser, 2009). Nearly all individuals that are persistently colonized with *H. pylori* will experience chronic gastritis, however, in a subset of individuals, *H. pylori* infection results in more severe disease outcomes including peptic and duodenal ulcers, neoplasia, dysplasia, B-cell lymphoma of mucosal-associated lymphoid tissue (MALT lymphoma), and invasive gastric adenocarcinoma (Cover and Blaser, 2009). For successful colonization of the host pathogenic bacteria must sense subtle changes in their environment, and rapidly respond with alterations in their transcriptional profile and *H. pylori* is no exception to this paradigm (Sharma et al., 2010). These environmental changes include the low pH characteristic of the gastric niche, alterations in nutrient availability including divalent cations, fluctuations in osmolarity, and the presence of the human immune system (Algood and Cover, 2006; de Bernard and Josenhans, 2014; Haley and Gaddy, 2015).

Transition metals participate in a multitude of critical biological processes, and are incorporated into numerous metalloproteins; making them a vital nutrient for all living organisms (Foster et al., 2014). The human host exploits this requirement for metals by restricting bacterial access to them in a dynamic process termed nutritional immunity (Hood and Skaar, 2012; Diaz-Ochoa et al., 2014). It is postulated that access to these nutrients within the gastric niche is incredibly variable, and therefore *H. pylori* likely experiences periodic abundances of critical metals followed by episodes of extreme depletion mediated by alterations in environmental pH, which can influence metal solubility, and expression of host derived metal-binding proteins such as the calgranulin proteins (calprotectin and S100A12) and iron-binding proteins like lactoferrin (Belzer et al., 2007; Senkovich et al., 2010; Gaddy et al., 2014; Haley et al., 2015). Much of the infectious potential of *H. pylori* is dependent on detecting fluctuations in metal availability and external ion concentrations, and responding with the expression of virulence factors and metal acquisitions systems (Danielli and Scarlato, 2010). For example, the cytotoxin-associated gene A (CagA) and the vacuolating cytotoxin (VacA) are both implicated in perturbing host cell iron trafficking, and both of these toxins are critical for host persistence (Salama et al., 2000; Tan et al., 2011). Detecting alterations in environmental metal quantities allows *H. pylori* to appropriately respond to changes in the host environment while simultaneously ensuring that its repertoire of metalloenzymes, which are required for essential processes within the cell, remain adequately metallated (Ge et al., 2013; Diaz-Ochoa et al., 2014). Many prokaryotes sense and respond to metals via two-component system (TCS) and signal-transduction networks (Silver and Walderhaug, 1992; Groisman, 2001; Eguchi and Utsumi, 2008). The typical *H. pylori* genome encodes remarkably few TCS, indicating metal sensing and response is likely done in novel ways (Panthel et al., 2003). Furthermore, it is likely that there are redundant and overlapping mechanisms that govern these regulons (Table 1). In this review we will highlight the complex regulatory networks, mediated through *trans*-regulatory elements including transcriptional regulators (TRs) and transcription factors, utilized by *H. pylori* to sense and react to subtle changes in extracellular metal concentrations within the human stomach.

**Table 1:** **Summary of genes regulated by metals and their corresponding regulatory mechanisms and references to the associated publication demonstrating these interactions**.

**Gene**	**Metal associated with regulation**	**Regulation mechanism**	**References**
fecA1	Iron	Fe-Fur (repression)	Ernst et al., 2005a; Danielli et al., 2006, 2009; Pich et al., 2012
fecA2	Iron	Fe-Fur (repression)	Fassbinder et al., 2000; Ernst et al., 2005a; Danielli et al., 2006, 2009; Pich et al., 2012
frpB1	Iron	Fe-Fur (repression)	Delany et al., 2001; Ernst et al., 2005a; Danielli et al., 2006; Pich et al., 2012
feoB	Iron	Fe-Fur (repression)	Ernst et al., 2005a
flaB	Iron	Fe-Fur (activation)	Danielli et al., 2006
fliY	Iron	Fe-Fur (activation)	Danielli et al., 2006
flgK	Iron	Fe-Fur (activation)	Danielli et al., 2006
cheA	Iron	Fe-Fur (activation)	Danielli et al., 2006
exbBD-tonB	Iron, Nickel, Copper	Fe-Fur (repression), Ni-NikR (repression), Copper (induction)	Contreras et al., 2003; Danielli et al., 2006
frpB4	Iron, Nickel	Fe-Fur (repression), Nik-R (repression)	Danielli et al., 2006; Ernst et al., 2006; Romagnoli et al., 2011
frpB2	Iron, Nickel	Fe-Fur (repression), Ni-NikR (repression)	Danielli et al., 2006; Muller et al., 2011
fecD	Iron	Fe-Fur (repression)	Danielli et al., 2006
yaeE	Iron	Fe-Fur (repression)	Danielli et al., 2006
pdxJ	Iron	Fe-Fur (repression)	Pich et al., 2012; Carpenter et al., 2013
pdxA	Iron	Fe-Fur (repression)	Carpenter et al., 2013
amiE	Iron	Fe-Fur (repression)	Pich et al., 2012
Hpn2	Iron, Nickel	Fe-Fur (repression), Ni-NikR (activation)	Contreras et al., 2003; Danielli et al., 2006
c553	Iron	Apo-Fur (repression)	Carpenter et al., 2013
hydAB^*^	Iron, Nickel	Apo-Fur (repression), Ni-NikR (repression)	Contreras et al., 2003; Carpenter et al., 2013
serB	Iron	Apo-Fur (repression)	Carpenter et al., 2013
pfr^*^	Iron, Copper, Zinc, Nickel, Manganese	Apo-Fur (repression), Fur-dependent (repression)	Waidner et al., 2005; Danielli et al., 2006; Carpenter et al., 2013; Zhao and Drlica, 2014
cagA	Iron	Fe-Fur (activation), apo-Fur (repression)	Odenbreit et al., 2000; Pich et al., 2012
oorDABC^*^	Iron	Fe-Fur (activation)	Gancz et al., 2006
ribBA	Iron	Fe-Fur (repression)	Worst et al., 1998; Fassbinder et al., 2000
sodB	Iron	Apo-Fur (repression)	Bereswill et al., 2000; Carpenter et al., 2013
fecA3	Nickel, Iron	Ni-NikR (repression), Fe-Fur (repression)	Ernst et al., 2006; Danielli et al., 2009
ceuA	Nickel	Ni-NikR (repression)	Muller et al., 2011
nixA	Nickel	Ni-NikR (repression)	Muller et al., 2011
nikR	Nickel	Ni-NikR (repression)	Muller et al., 2011
hspA	Nickel	Ni-NikR (activation)	Contreras et al., 2003
hpn	Nickel	Ni-NikR (activation)	Contreras et al., 2003
ureA	Nickel	Ni-NikR (activation), Mua (repression)	Benoit and Maier, 2011
fliS	Copper	Copper (activation)	Waidner et al., 2002
rlmA	Copper	Copper (activation)	Waidner et al., 2002
nadC	Copper	Copper (activation)	Waidner et al., 2002
trpA	Copper	Copper (activation)	Waidner et al., 2002
HP1255	Copper	Copper (activation)	Waidner et al., 2002
HP1516	Copper	Copper (activation)	Waidner et al., 2002
HP0733	Copper	Copper (activation)	Waidner et al., 2002
HP0994	Copper	Copper (activation)	Waidner et al., 2002
hpylM	Copper	Copper (activation)	Waidner et al., 2002
nadC	Copper	Copper (activation)	Waidner et al., 2002
proC	Copper	Copper (activation)	Waidner et al., 2002
crdAB^*^	Copper	CrdRS (activation)	Waidner et al., 2005
czcAB^*^	Copper, Zinc	CrdRS (activation), Zinc (activation)	Waidner et al., 2005; Stähler et al., 2006
copAB	Copper	CopP (activation)	Waidner et al., 2005
cznABC	Zinc	Zinc (activation)	Stähler et al., 2006

* *Indicates gene(s) located in an operon that are co-regulated*.

## Iron

**Figure 1:**
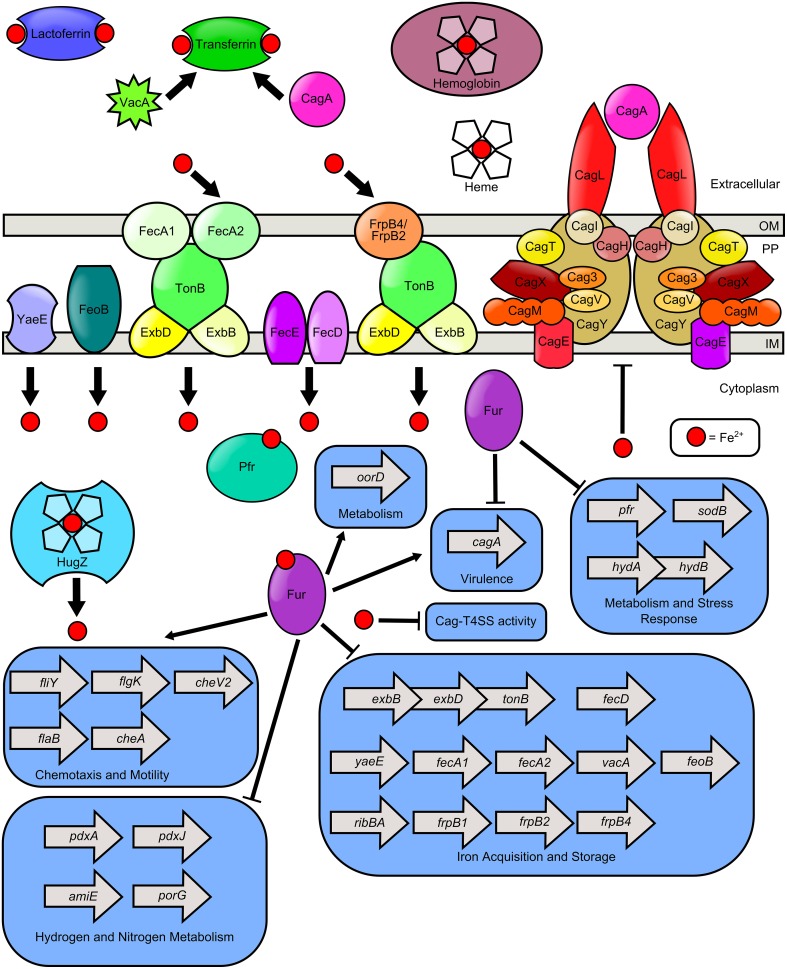
**Model of iron transport and iron-dependent gene regulation in ***H. pylori***.** *H. pylori* can use numerous sources of nutrient iron including hemoglobin, holo-lactoferrin, holo-transferrin, and heme, which is degraded in the cytoplasm by the heme oxygenase HugZ. Host cell transferrin can be trafficked to the apical cell surface by the cytotoxins VacA and CagA, produced by *H. pylori*. FecA1, FecA2, FrpB4, and FrpB2 are implicated as outer membrane iron receptors which transport iron through the TonB-ExbB-ExbD complex. FeoB and YaeE are putative permeases which shuttle iron from the periplasm, across the inner membrane to the cytoplasm. The FecD/FecE system also shuttles iron from the periplasm to the cytoplasm. Once in the cytoplasm, iron binds to Fur and promotes expression of the *fliY, flgK, flab*, and *cheA* genes which encode flagellar and chemotactic components. Fe-Fur also represses the expression of numerous genes involved in iron homeostasis (including *exbB-exbD-tonB, fecD, yaeE, fecA1, fecA2, vacA, feoB, ribBA, frpB1, frpB2, frpB4*) and metabolism of hydrogen or nitrogen (including *pdxA, pdxJ, amiE*, and *porG*). Furthermore, apo-Fur represses *pfr, sodB, hydAB*, and *cagA* expression. Iron is stored by bacterial ferritin (Pfr) and free cytoplasmic iron can repress elaboration of Cag-T4SS pili and activity of the T4SS.

The use of iron in a number of critical metabolic pathways including electron transport, DNA replication, and amino acid synthesis, as well as its role as a cofactor within iron sulfur clusters and heme, makes it a necessity for nearly every living organism including *H. pylori* (Becker and Skaar, 2014). The human body exploits this need for iron by limiting access to this critical micronutrient through nutritional immunity (Cassat and Skaar, 2013). Iron is bound within host molecules such as lactoferrin, transferrin, heme, hemoglobin, and haptoglobin, making it relatively unavailable to pathogens (Yen et al., 2011; Haley and Skaar, 2012). Within the gastric niche *H. pylori* has access to host dietary iron, however, it must compete with the host for this limited nutrient (Figure 1). In conditions of restricted iron availability, *H. pylori* can utilize alternate sources of nutrient iron including hemoglobin, transferrin, heme, and lactoferrin (Dhaenens et al., 1999; Senkovich et al., 2010). Consequently, *H. pylori* responds to iron limitation by upregulating an arsenal of molecular pathways devoted to diverse iron acquisition functions; therefore, the ability to detect and respond to environmental iron concentrations is critical to the survival of *H. pylori*.

The number of TRs utilized by *H. pylori* is relatively small for a genome of its size (~1600 kb), and consequently the TRs of *H. pylori* display a more diverse array of functions than their counterparts in other bacteria (Pich et al., 2012; Troxell and Hassan, 2013). The broad activity of *H. pylori* TRs is best exemplified by the ferric uptake regulator, Fur. Canonically Fur binds its ferrous iron cofactor to form holo-Fur (Fe-Fur) which then mediates Fur binding to conserved DNA sequences, specifically a 7-1-7 motif with dyad symmetry (5′-TAATAATnATTATTA-3′) within the promoter region of regulated genes resulting in the repression of their expression (Pich et al., 2012). While holo-Fur repression is the best characterized Fur mediated regulation, it is not the only way in which Fur modifies transcriptional rates. Specifically, within *H. pylori*, holo-Fur has been shown to function as an activator, and apo-Fur, which is not bound to an iron cofactor, has also been shown to regulate gene transcription (Gancz et al., 2006; Carpenter et al., 2013). This unique flexible transcriptional regulation facilitated by Fur indicates that Fur functions as a global regulator within *H. pylori*; therefore, minute alterations in iron availability can lead to significant changes in the transcriptome of *H. pylori*.

### Holo-fur

Many of the *H. pylori* genes which are regulated by Fur in the traditional manner are involved in iron acquisition. This allows *H. pylori*, upon experiencing iron starvation, to upregulate a complex network of proteins that facilitate the acquisition and trafficking of this vital nutrient. For example, upon experiencing iron restriction Fur is no longer able to bind to the promoter regions of the genes encoding the high affinity iron transporters *fecA1, fecA2, frpB1*, and *feoB*, thus resulting in a dramatic increase in transcriptional rates of these genes and subsequent iron import into the cell (Delany et al., 2001; Ernst et al., 2005a; Danielli et al., 2009). In iron replete conditions, Fe-Fur represses a variety of iron transport genes including the *exbB-exbD-tonB* operon, *frpB4*, and *frpB2*, which encode outer membrane iron transporters, *fecD*, and *yaeE*, which encode inner membrane iron permeases (Fassbinder et al., 2000; Danielli et al., 2006). The Fur-regulon also includes genes which encode proteins involved in nitrogen and hydrogen metabolism, including *amiE*, the gene encoding pyruvate ferrodoxin oxidoreductase (*porG*), and genes implicated in pyridoxal phosphate biosynthesis (*pdxJ* and *pdxA*) (Gancz et al., 2006; Carpenter et al., 2013). Together, these findings indicate that *H. pylori* exploits Fur to regulate and enable metabolic flexibility which helps the cell circumnavigate the stress imposed by changes in iron availability. In addition to increasing expression of iron transporters upon iron starvation *H. pylori* also increases binding to the host chelating proteins lactoferrin, and transferrin presumably by upregulating expression of the receptors for these proteins (Senkovich et al., 2010). Thus, the Fur-mediated iron regulation of these iron acquisition systems enables *H. pylori* to rapidly respond to the host-imposed iron limitation by increasing intracellular iron import.

An additional strategy employed by *H. pylori* to alleviate iron starvation is to increase the biosynthesis of flavins, which are used within ferric iron reductases to mediate the reduction of extracellular iron within iron-containing compounds. Reduction of iron to its more soluble ferrous form increases the concentration of available iron while decreasing the affinity of the iron for some host ligands (Worst et al., 1998). Expression of the enzyme responsible for the initial rate limiting step in riboflavin synthesis, RibBA, is repressed by holo-Fur. Consequently, iron reductases are indirectly Fur-regulated, such that upon encountering iron restriction, *H. pylori* increases transcription of *ribBA* resulting in an increase in flavin production that can then be used by the ferric iron reductases (Worst et al., 1998).

Iron-bound Fur has also been shown to directly activate gene expression including *fliY, flgK, flaB*, and *cheA* which encode flagellar and chemotactic components (Danielli et al., 2006). Additionally, holo-Fur has been shown to activate the *oorDABC* operon which encodes for a 2-oxoglutarate oxidoreductase, an enzyme that catalyzes the formation of succinyl-CoA, a major intermediate in carbon metabolism (Gilbreath et al., 2012). This non-canonical activity of Fur, which is commonly thought to be directly involved in gene repression, highlights the flexibility of this molecule within *H. pylori*; a characteristic that likely aids in persistence in the gastric niche.

### Apo-fur

While iron acquisition is critical for *H. pylori* colonization, excessive cellular levels of iron can be detrimental to the cell as it can lead to the formation of reactive oxygen species through the Fenton reaction. Reactive oxygen species can lead to DNA, protein, and lipid membrane damage (Aguirre and Culotta, 2012; Zhao and Drlica, 2014). To mitigate this damage, *H. pylori* increases expression of the bacterial ferritin Pfr, an iron storage protein, in response to high iron levels. Iron regulation of *pfr* is facilitated by Fur, however, this regulation occurs through non-canonical Fur regulation such that apo-Fur represses *pfr* transcription (Bereswill et al., 2000; Carpenter et al., 2013). An additional mechanism for coping with oxidative stress induced by high iron levels is through the activity of superoxide dismutases as it catalyzes the conversion of superoxide species into oxygen and hydrogen peroxide (Ernst et al., 2005b). Not surprisingly, the only identified superoxide dismutase in *H. pylori*, SodB, is transcriptionally regulated by Fur, such that apo-Fur binds to the promoter region of *sodB*, occluding RNA polymerase binding (Ernst et al., 2005b). The combinatorial result of apo-Fur regulation of both *sodB* and *pfr* is *H. pylori* can appropriately respond to high iron levels with a set of proteins designed to alleviate iron toxicity. Interestingly, apo-Fur has been shown to repress *hydABCDE*, an operon which encodes a Ni/Fe hydrogenase, indicating that apo-Fur is also implicated in the regulation of hydrogen metabolism in *H. pylori*. It is also postulated that apo-Fur-dependent repression of genes (including *sodB* and those encoded in the *hydABCDE* operon) evolved because their protein products utilize iron as a cofactor, and repression by apo-Fur ensures these proteins are produced only when sufficient levels of that cofactor are available (Ernst et al., 2005b).

The iron restrictive nature of the host has led to many bacterial pathogens coordinating the production of virulence factors to the detection of low iron availability. *H. pylori* encodes two important virulence factors, the VacA, and the cytotoxin-associated gene A (CagA) toxin (Cover and Peek, 2013). VacA is secreted via the Type V autotransporter pathway (Fischer et al., 2001; Letley et al., 2006). VacA causes numerous changes in host cells including vacuolation, depolarization of the membrane potential, permeabilization of epithelial monolayers, disruption of lysosomes, inhibition of T-cell activation and proliferation, and ultimately leads to cell death via programmed necrosis (Satin et al., 1997; Szabò et al., 1999; Sundrud et al., 2004; Torres et al., 2007; Radin et al., 2011). CagA is secreted via a type IV secretion system (T4SS), which is encoded within the *cag* pathogenicity island (Tummuru et al., 1995). Upon translocation into host cells, CagA is phosphorylated, and induces a cascade of changes within the host cell ultimately leading to changes in immune signaling and cell morphology (Odenbreit et al., 2000). Interestingly, one effect of CagA translocation into host cells is the marked alteration in host cell polarity, which results in apical release of transferrin (Tan et al., 2011). VacA is also implicated in perturbing transferrin trafficking in epithelial monolayers to the apical cell surface (Tan et al., 2011). This apically localized transferrin is believed to be used as an iron source by *H. pylori*, thus allowing this bacterium to modify the host environment and create a more hospitable replicative niche. Given its role in immune modulation and iron acquisition, it is not surprising that Fur has been implicated as a potential regulator of *cagA* expression (Pich et al., 2012; Vannini et al., 2014). Along with these observations, our work has shown that conditions of low nutrient iron availability induce expression of the Cag-T4SS pili at the host-pathogen interface, and also enhance the activity of the Cag-T4SS (Noto et al., 2013, 2014; Haley et al., 2014). Additionally, transcription of *cagA* has shown to be upregulated in iron-replete conditions suggesting Fe-Fur activation of *cagA* (Pich et al., 2012). These seemingly contradictory findings suggest an additional, as yet unknown, environmental signal modifies the Fur-mediated regulation of *cagA* activity. Thus, these observations reveal that *H. pylori* toxin secretion is regulated by iron and plays a role in iron homeostasis as well as suggests a role for additional signals to influence iron-mediated regulation of *cagA*.

### Importance of fur *in vivo*

The importance of sensing environmental iron levels, and responding with global transcriptional changes, is underscored by the colonization defect of the *H. pylori* Δ*fur* strain within a Mongolian gerbil animal model (Gancz et al., 2006; Miles et al., 2010). Interestingly, the colonization defect of the Δ*fur* strain as compared to the wild-type strain was most severe early in infection with a >50 fold decrease in the number of CFU/g stomach tissue recoverable at day 3 post infection (Miles et al., 2010). Importantly, this initial decrease in bacterial burden dissipates over time and by day 14 no discernable differences between the wild-type and Δ*fur* strain could be detected, indicating that Fur-mediated transcriptional regulation is most critical early in the infection process (Miles et al., 2010). Furthermore, while *H. pylori* has been shown to localize to both the corpus and antrum of the stomach the highest level of bacterial burden is typically associated with the antral region. This localization pattern is maintained within a gerbil infection model where it has been shown that in the absence of Fur, *H. pylori* preferentially colonizes the corpus as opposed to the antrum (Miles et al., 2010). The precise mechanism for this aberrant distribution within the Δ*fur* strain may be due to transcriptional changes of *cheV2*, a Fur-regulated gene shown to be involved in chemotaxis (Miles et al., 2010).

## Nickel

**Figure 2:**
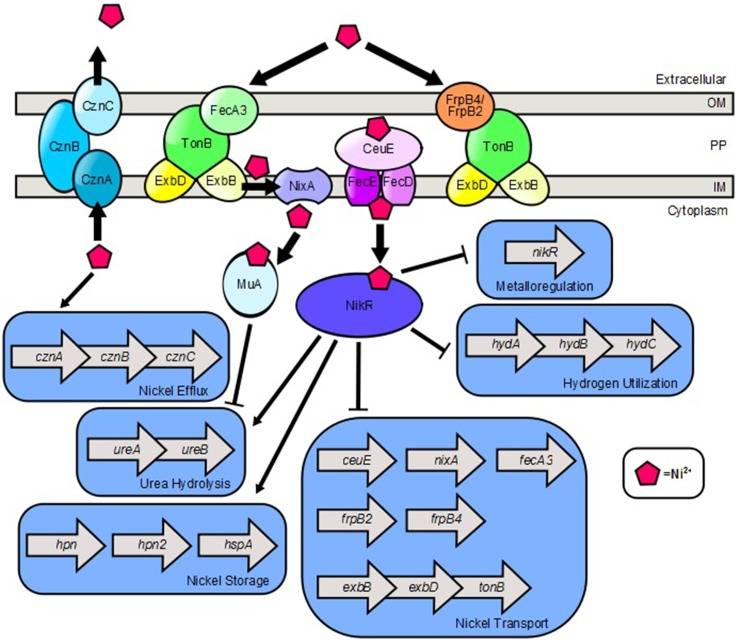
**Model of nickel transport and nickel-dependent gene regulation in ***H. pylori*****. FecA3, FrpB4, and FrpB2 are implicated as outer membrane nickel receptors which transport nickel through the TonB-ExbB-ExbD complex. NixA is a nickel permease which shuttles nickel from the periplasm, across the inner membrane to the cytoplasm. CeuE is a periplasmic nickel binding protein that is believed to shuttle nickel through the FecD/FecE system to the cytoplasm. Once in the cytoplasm, nickel promotes expression of the *cznABC* operon which encodes a nickel efflux pump. Nickel binds to NikR which promotes expression of genes involved in nickel storage and urea hydrolysis. Ni^2+^-NikR represses its own coding, as well as nickel transport genes and hydrogen utilization genes. Nickel also binds to the Mua protein which represses transcription of genes encoding urease subunits.

The transition metal nickel plays an important role in *H. pylori* physiology and pathogenesis (Figure 2). Nickel is a cofactor for two critical metalloenzymes, urease and [NiFe]-hydrogenase (de Reuse et al., 2013; Sydor et al., 2014). The former catalyzes the generation of ammonia and bicarbonate from urea, a process that provides a protective increase in pH, enabling the bacterium to withstand the acidic environment of the stomach (Evans et al., 1991; Hawtin et al., 1991; Maier, 2005; Benoit and Maier, 2008). The latter, enables the bacterium to utilize hydrogen gas as an energy source within the gastric niche. Because nickel is required for the activity of these important enzymes, nickel acquisition is a nutritional requirement for *H. pylori* (de Reuse et al., 2013). However, accumulation of high concentrations of intracellular nickel can be toxic to the cells (Benoit and Maier, 2008). Thus, nickel acquisition and distribution are tightly controlled by multiple features including transport, storage, and efflux.

The master regulator for nickel homeostasis is a nickel-responsive TR referred to as, NikR (Dosanjh and Michel, 2006). *H. pylori* NikR can act as either a repressor or an activator, although the precise mechanism by which nickel-sensing affects the DNA-binding capacity of NikR remains largely unknown, however a putative NikR- DNA-binding consensus sequence has been identified (TATWATT-N_11_-AATWATA) (Ernst et al., 2006). The NikR-regulon includes multiple genes involved in metal homeostasis, hydrogen utilization, and acid response with chronological hierarchy (Jones et al., 2014). NikR represses transcription of HPG27_1499 (*ceuE*) and HP1077 (*nixA*), genes which encode a periplasmic nickel transporter and an inner membrane nickel permease, respectively, HPG27_866, a gene encoding FrpB2, (Muller et al., 2011) which contributes to the accumulation of cell-associated nickel, HP1339, the first gene in the operon which encodes ExbB/ExbD/TonB energy transport system, and the *hydABC* locus which encodes proteins involved in hydrogen utilization (Contreras et al., 2003). NikR also represses *fecA3* and *frpB4*, which encode a putative outer membrane receptors for nickel (Ernst et al., 2006; Romagnoli et al., 2011). Furthermore, NikR is an autoregulator that binds to its own intergenic region and represses expression of the *nikR* locus in conditions of excess nickel (Contreras et al., 2003). Interestingly, Ni^2+^-NikR also induces transcription of numerous genes including *hpn, hpn2*, and *hspA* which are involved in nickel storage, and the *ureA* locus (HP0073) in which the first gene in the operon encodes the subunits of the urease enzyme (Contreras et al., 2003). Urease is an important nickel-containing dodecameric enzyme that buffers the cytoplasm and the periplasm of *H. pylori* during colonization of the acidic gastric niche (Khan et al., 2009). Urease is the most abundant enzyme in *H. pylori* cells, accounting for almost 10% of the total cellular protein content (Benoit and Maier, 2011). Urease expression is critical for early colonization and virulence in the vertebrate host (Eaton et al., 1991). The urease complex is comprised of subunits organized into two transcriptional units, *ureAB* and *ureIEFGH*, which produces three transcripts, *ureAB, ureIEFGH*, and *ureABIEFGH* (Akada et al., 2000). These transcripts are induced, as part of the acid stress response through the phosphorylation of the ArsRS TCS (Pflock et al., 2005). Conversely, these transcripts are repressed at neutral pH by the unphosphorylated ArsRS system via a cis-encoded antisense small RNA to *ureB* (Wen et al., 2011). In addition to the canonical NikR-mediated activation of urease expression in response to nickel availability, recent evidence suggests that the HP0868 (Mua) protein can repress expression of *ureA* under conditions where nickel is abundant in the intracellular compartment (Benoit and Maier, 2011).

Nickel transport into the cell is facilitated through FrpB4, FrpB2, or FecA3, outer membrane receptors involved in nickel uptake work in tandem with ExbB/ExbD/TonB machinery to import nickel across the outer membrane (Schauer et al., 2007). NixA, an inner membrane protein then facilitates nickel transport from the periplasm to the cytoplasm. Alternatively, nickel can be transported by the periplasmic transporter CeuE, which likely works cooperatively with the inner membrane FecD/E ABC transporter, (Figure 2) which has been implicated in *H. mustelae* nickel and cobalt acquisition (Stoof et al., 2010). As nickel concentration increases in the cytoplasm, *H. pylori* induces expression of the *cznABC* loci, which encode components of a putative cobalt, zinc, and nickel efflux pump. This efflux system is critical for resistance to nickel stress and colonization of the vertebrate host (Stähler et al., 2006). Although *H. pylori* does not seem to have a strict nutritional requirement for nickel to survive (Testerman et al., 2006), it is clear that nickel sensing and nickel homeostasis are important for *H. pylori* persistence in the human gastric niche as underscored by the significant colonization defect of *H. pylori* strains lacking a functional *nikR* gene as compared to wildtype *H. pylori* strains (Bury-Mone et al., 2004).

## Copper

**Figure 3:**
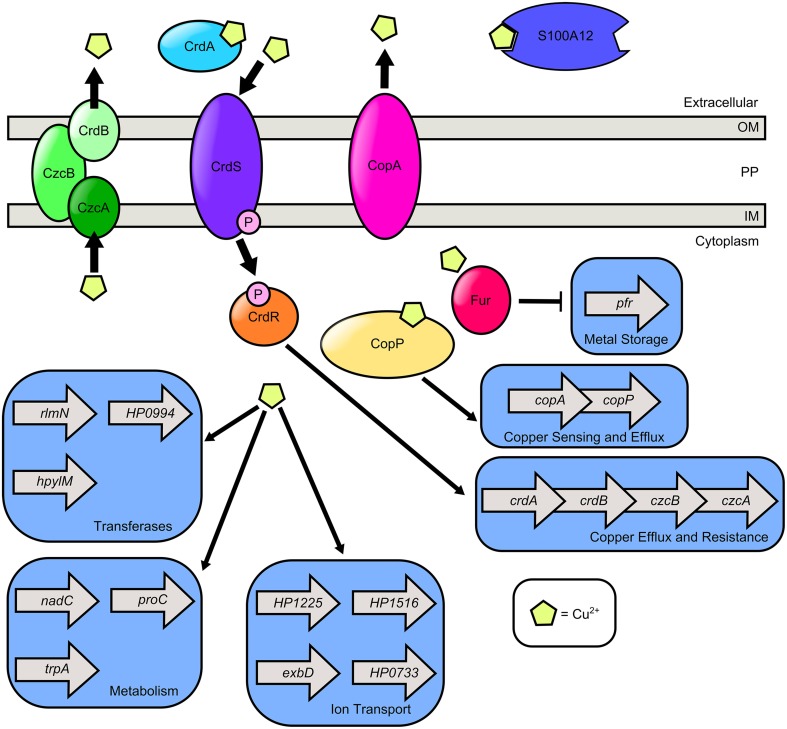
**Model of copper transport and copper-dependent gene regulation in ***H. pylori*****. *H. pylori* secretes CrdA, a copper-binding protein that facilitates copper resistance. Extracellular copper is sensed by the CrdS sensor kinase which phosphorylates the cognate response regulator CrdR, an activator of copper efflux and resistance expression. S100A12 is a host protein that can bind copper as well as zinc. It is hypothesized that cytoplasmic copper levels are sensed by Cu^2+^-CopP which upregulates the *copAP* operon encoding the CopA copper resistance protein and the CopP regulator. In the presence of copper, cytoplasmic levels of ferritin (Pfr) are reduced in a Fur dependent manner. Copper exposure promotes expression of the methyltransferase (*rlmN*), the nucleotidyl transferase (*HP0994*), and the DNA-methyltransferase (*hpylM*). Copper induces transcription of nicotinate-nucleotide pyrophosphorylase (*nadC*), pyrroline-5-carboxylate reductase (*proC*), and tryptophan synthase (*trpA*). Copper promotes transcription of genes involved in ion transport including *exbD, crcB* which encodes a camphor resistance protein involved in fluoride ion transport (*HP1225*), a putative ABC-transporter (*HP1516*), and a GTPase with putative ABC-transporter activity (*HP0733*). *H. pylori* encodes two Cu^2+^ efflux systems, the CrdB-CzcAB complex and the CopA system.

In the gastric environment, dietary copper intake can exceed 1 mg, indicating copper is present in micromolar concentration at the lumen of the stomach (Barceloux, 1999). Copper can also be sequestered by S100A12 (Haley et al., 2015). Copper is gaining increasing recognition as an important component of macrophage-mediated antimicrobial activity (Wolschendorf et al., 2011; Johnson et al., 2015; Neyrolles et al., 2015). Macrophages exploit copper toxicity to poison bacteria within the phagosome presumably by inducing Fenton-like reactions which produce hydroxyl radicals (Pham et al., 2013; Neyrolles et al., 2015). Conversely, bacteria also utilize copper as a cofactor for oxidases, electron transport proteins, and hydroxylases. *H. pylori* encodes both menaquinone-6 and a cbb3-type cytochrome-c oxidase, which harbors a heme-copper binuclear center similar to the cytochrome aa3-type oxidase (Nagata et al., 1996). This is likely the terminal oxidase in the *H. pylori* respiratory chain, and thus, copper is a critical cofactor for respiration. Copper promotes *H. pylori* colonization of mucosal surfaces and also acts as a chemotactic repellant for bacterial cell motility (Montefusco et al., 2013; Sanders et al., 2013). Thus, *H. pylori* must balance the need for copper as a respiration cofactor and the importance of protection against copper toxicity (Figure 3).

Exposure to copper alters the *H. pylori* transcriptional profile in numerous ways. In the presence of copper and other metals, the cytoplasmic levels of Pfr are reduced in a Fur dependent manner, suggesting Fur-mediated repression of *pfr* transcription in an undefined mechanism (Bereswill et al., 2000). This is likely to facilitate survival under conditions of metal stress. Copper is also associated with upregulation of numerous genes involved in a variety of cellular responses including motility, *fliS*- encoding a putative flagellin chaperone, ion transport, *exbD* which encodes an energy transport protein involved in iron and nickel acquisition, *crcB*, which encodes camphor resistance protein involved in fluoride ion transport (*HP1225*), a putative ABC-transporter (*HP1516*), and a GTPase with putative ABC-transporter activity (*HP0733*); transferase activity (*rlmN- encoding methyltransferase*), the nucleotidyl transferase (*HP0994*), and the DNA-methyltransferase (*hpylM*) (Waidner et al., 2002). Copper upregulates transcription of genes involved in several metabolic pathways including nicotinate-nucleotide pyrophosphorylase (*nadC*), pyrroline-5-carboxylate reductase (*proC*), and tryptophan synthase (*trpA*). *H. pylori* also upregulates numerous genes involved in copper homeostasis such as *crdAB, czcAB*, and the *copAP* operon in response to copper stress (Waidner et al., 2002).

To evade copper toxicity, *H. pylori* has evolved elegant efflux strategies governed by copper sensing mechanisms. *H. pylori* employs a TCS *crdRS* encoding a sensor kinase (CrdS) which phosphorylates a cognate response regulator (CrdR) in the presence of copper (Waidner et al., 2005). CrdR induces expression of *crdAB* and *czcAB*, which encode a secreted copper resistance protein (CrdA) and a copper efflux complex comprised of CrdB, CzcB, and CzcA. *H. pylori* also utilizes the *copAP* operon, which encodes a cytoplasmic copper-binding regulator homologous to CopZ from *E. coli* (CopP) that promotes expression of CopA, a protein which promotes resistance to copper (Beier et al., 1997). Taken together, these results reveal that tight molecular mechanisms of gene regulation have evolved in the human pathogen *H. pylori* in response to changes in copper availability, and that these regulatory events are critical for adaptation to the gastric environment.

## Zinc

**Figure 4:**
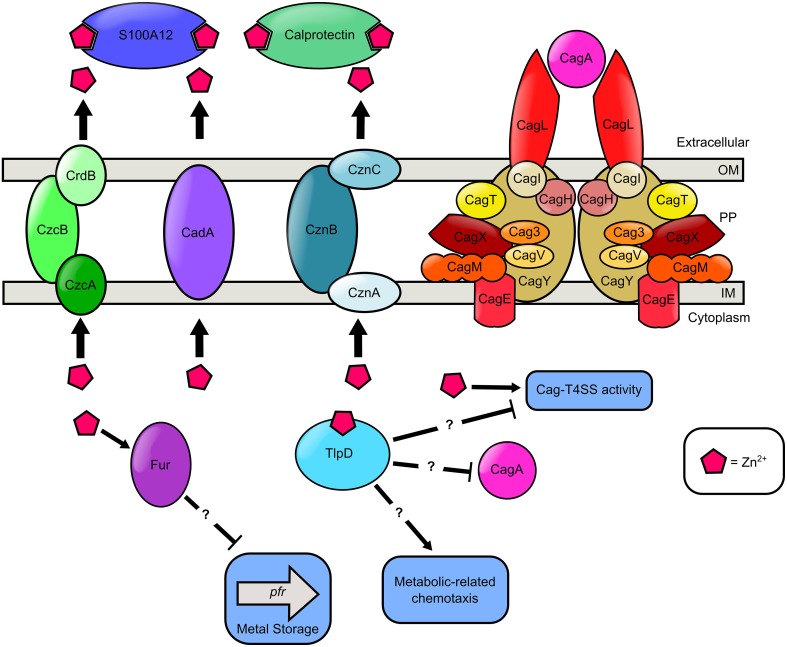
**Model of zinc transport and zinc-dependent regulation of chemotaxis, metal storage, and virulence in ***H. pylori*****. Extracellular zinc is bound by the S100A-family host antimicrobial proteins, calprotectin and S100A12, produced by innate immune cells. Cytoplasmic zinc binds the cytoplasmic chemotactic receptor TlpD. High cytoplasmic levels of zinc results in a reduction in Pfr expression in a Fur dependent manner. TlpD putatively participates in metabolic-related chemotaxis, CagA repression, and repression of the Cag-T4SS activity. Nutrient zinc is required for Cag-T4SS pilus deployment and activity. In the presence of excess zinc, *H. pylori* utilizes zinc efflux pumps as a detoxification strategy. *H. pylori* encodes three Zn^2+^ efflux systems, the CrdB-CzcAB complex, the CadA transporter, and the CznABC system. Question marks “?” indicate proteins involved in zinc-responsive changes in cell biology are influencing regulation in an undefined manner.

Zinc is a micronutrient required for all forms of life, including *H. pylori* (Testerman et al., 2006; Kehl-Fie and Skaar, 2010). Zinc availability is controlled tightly at the host-pathogen interface by host S100A-family proteins including calprotectin, which participate in nutritional immunity via transition metal sequestration (Kehl-Fie and Skaar, 2010). Coinciding with this, increased severity of *H. pylori*-induced gastric inflammation is inversely correlated with zinc concentrations within the gastric mucosa (Sempértegui et al., 2007). Conversely, humans consume large quantities of transition metals such as zinc, so it is likely that *H. pylori* is exposed to micromolar concentrations of zinc at the lumen of the stomach (Stähler et al., 2006). To persist in the gastric environment, *H. pylori* must quickly adapt to variations in zinc availability (Figure 4). One way that *H. pylori* senses and responds to metal signals in the gastric niche is through chemotaxis, the sensing of and response to a chemical signal. Zinc is a chemotactic attractant for *H. pylori*, whereas nickel is a repellent (Sanders et al., 2013). *H. pylori* possesses four chemotactic receptors, TlpABCD, which participate in environmental sensing (Lertsethtakarn et al., 2011; Rader et al., 2011). TlpD is required for colonization of the antrum of the stomach and is important for bacterial motility (Rolig et al., 2012). In *H. pylori*, chemotaxis has been shown to be important for the elicitation of an inflammation response during infection (McGee et al., 2005; Williams et al., 2007). Interestingly, TlpD has a zinc-binding domain which could participate in its chemotactic activity and zinc-sensing (Draper et al., 2011). A *tlpD* mutant elicited more IL-8 from epithelial cells and produced more CagA compared to the WT parental strain, although the mechanism of this regulation remains obscure (Behrens et al., 2013). Recent work indicates that zinc sequestration by host antimicrobial proteins such as calprotectin (S100A8/A9 heterodimer) or calgranulin C (S100A12 homodimer) represses the elaboration of the extracellular pilus associated with the *H. pylori cag*-type IV secretion system (Cag-T4SS) and its associated activity (Gaddy et al., 2014; Haley et al., 2015). The Cag-T4SS is a macromolecular machine that is responsible for translocation of the oncogenic effector cytotoxin CagA. Strains of *H. pylori* that possess CagA are associated with increased risk of disease outcomes including gastritis and gastric cancer (Blaser et al., 1995). Zinc is required for Cag-T4SS pilus formation, CagA translocation, activation of nuclear factor kappa-β, and IL-8 secretion by host cells (Gaddy et al., 2014). Together, these results reveal that zinc is an important global signal that regulates virulence.

In addition to being an important environmental signal, high levels of zinc are toxic to bacterial cells (Braymer and Giedroc, 2014). There is growing evidence that the host innate immune system exploits zinc and copper to poison bacteria trapped within the macrophage phagosome (Neyrolles et al., 2013). To evade zinc toxicity, bacterial pathogens have evolved efflux strategies to transport zinc to the extracellular space (Braymer and Giedroc, 2014). *H. pylori* has three zinc transport systems that participate in zinc resistance including CadA, the CrdB-CzcAB complex, and the CznABC system (Waidner et al., 2002, 2005; Stähler et al., 2006). *H. pylori* mutants harboring inactivation in the coding region of the CadA transition metal ATPase exhibit increased sensitivity to both cadmium and zinc (Herrmann et al., 1999). *H. pylori cznA, cznB*, and *cznC* mutants have increased sensitivity to zinc toxicity, and also exhibit elevated cytoplasmic levels of zinc, indicating zinc ions are trapped within the cell (Stähler et al., 2006). These mutants are defective for colonization of rodent models, implicating the CznABC efflux pump as an important virulence determinant (Stähler et al., 2006). Furthermore, the *H. pylori* genome encodes a redundant CrdB-CzcAB complex purported to be involved in zinc efflux (Stähler et al., 2006). Interestingly, while it has been unequivocally demonstrated that zinc is a vital nutrient for *H. pylori* survival no zinc import system has been identified. One possible explanation for the absence of a zinc uptake system is that many metal import systems are promiscuous and facilitate the acquisition of multiple metals. It is therefore feasible that the nickel import systems also facilitate zinc acquisition. Additionally, the promiscuity of metal influx structures may hinder traditional genetic screening methods used to identify putative import proteins as the ablation of any one single gene will not result in a significant growth defect. These results indicate that *H. pylori* must maintain zinc homeostasis as a prerequisite for colonization of the host and persistence within the host to establish a chronic infection.

## Magnesium, manganese, and cobalt

While it is clear that magnesium, manganese, and cobalt are important cofactors for various bacterial proteins, the current depth of understanding regarding the role, regulation, and acquisition of these metals is limited relative to iron, nickel, zinc, and copper. Magnesium is required for the activity of several biochemical pathways in central metabolism, which are essential for bacterial growth and viability (Smith and Maguire, 1998). For example, *H. pylori* heavily relies on Mg^2+^ for phosphate metabolism, including the catabolism of phosphonates, which can function as both a vital source of phosphorous and as phosphorous storage (Ford et al., 2010). *H. pylori* is capable of degrading the phosphonate, phenylphosphonate, to use as a sole phosphate source. This catabolism was shown to be enhanced by the presence of exogenous MgCl^2+^ and inhibited upon the addition of the chelator EDTA (Ford et al., 2010). Mg^2+^ also plays a critical role in *H. pylori* phosphate metabolism as a metal cofactor within pyrophosphatase. Pyrophosphatases are ubiquitious enzymes that catalyze the interconversion of inorganic pyrophosphate and orthophosphate, and function as a catalyst within cellular bioenergetics, making this group of enzymes essential for life. The pyrophosphatases within *H. pylori* requires Mg^2+^ for enzymatic activity (Lee et al., 2007). In addition to its role as a cofactor within *H. pylori* phosphate metabolism, Mg^2+^ may also play an important role in *H. pylori* transcriptional regulation through the binding of a lower affinity site within the transcription factor NikR. Although NikR binds nickel and has been shown to be nickel responsive, a second binding site has been shown to require either Mg^2+^ or calcium for NikR promoter binding (Dosanjh et al., 2007). The importance of Mg^2+^ is underscored by the strict requirement of magnesium for *H. pylori* cell viability (Testerman et al., 2006). To satisfy this Mg^2+^ requirement *H. pylori* has evolved systems to transport this important metal. CorA, a distant homolog (15–20% sequence identity) of the *Salmonella enterica* serovar Typhimurium ZntB Zn^2+^ efflux transporter, is involved in manganese uptake in *H. pylori* (Wan et al., 2011). Isogenic *corA* mutants have diminished growth in the absence of an exogenous source of nutrient magnesium compared to the parental strain, and expression of the *H. pylori corA* locus *in trans* in an *E. coli corA* mutant enhanced magnesium toxicity compared to the *E. coli corA* mutant, demonstrating its role in magnesium transport (Pfeiffer et al., 2002). CorA is also implicated in cobalt and nickel transport, however, magnesium is the dominant substrate for this protein (Pfeiffer et al., 2002; Wan et al., 2011).

Manganese is an important nutrient for *H. pylori* due to its use as a cofactor within the pyrimidine metabolic pathway. Uridine monophosphate (UMP) kinase is a necessary enzyme for the synthesis of pyrimidine nucleotides. The UMP kinase in *H. pylori* is unique in its use of manganese as a cofactor as opposed to the canonical magnesium (Lee et al., 2010). Manganese also functions as a critical cofactor within the *H. pylori* phosphatidylserine synthase (PSS), an enzyme necessary for the biosynthesis of phospholipids. Specifically, PSS is responsible for the catalysis of the first committed step in the formation of the phospholipid phosphatidylethanolamine, a major component of the phospholipid membrane (Ge and Taylor, 1997). The importance of PSS and therefore manganese is highlighted by the inability to ablate the gene encoding PSS as it is essential for cell viability (Ge and Taylor, 1997).

Cobalt is gaining appreciation as an important micronutrient. Cobalt, among several other transition metals, can serve as a cofactor for HpyAV, a type II restriction-modification system in *H. pylori*. Type II restriction-modification endonucleases are highly conserved in prokaryotic organisms, but *H. pylori* is particularly rich with these, likely due to acquisition through natural competence. Type II restriction-modification endonucleases are implicated in phage resistance and transcriptional regulation of gene expression (Chan et al., 2010). Additionally, cobalt is utilized as a cofactor within the *H. pylori* arginase. Arginases are a group of enzymes that are responsible for catalyzing the conversion of L-arginine to L-ornithine. The ubiquitous use of arginases across multiple kingdoms underscores the importance of this enzyme in maintaining arginine homeostasis. The *H. pylori* arginase has been shown to be critical for acid protection *in vitro* and to play an important role during colonization. Many arginases utilize Mn^2+^ as a metal cofactor; however, the *H. pylori* arginase is unique in its use of Co^2+^ as a cofactor (Srivastava et al., 2011). To fulfill its need for cobalt *H. pylori* transports cobalt into the cell using the metal transporter CorA (Pfeiffer et al., 2002). Although, cobalt is an invaluable micronutrient at high quantities cobalt can exert cellular toxicity. To prevent metal toxicity *H. pylori* utilizes the metal ion efflux pump CadA which has specificity for Co (II), Cd (II), and Zn (II) (Herrmann et al., 1999).

## Conclusions

The human stomach is an incredibly dynamic and seemingly inhospitable environment for an invading prokaryote. Yet, it is within this environment that *H. pylori* establishes its replicative niche. To thrive in an environment of oscillating extremes including pH, immune assault and nutrient availability *H. pylori* alters its transcriptional profile through the intricate interplay of multiple transcriptional regulators. Transcriptional changes mediated through the sensing of metals enables *H. pylori* to detect changes in nutrient availability and deploy an impressive array of metal acquisition systems, store the corresponding influx of metals, avoid metal toxicity and circumnavigate the host immune assault. Many bacteria use metal sensing as a way to precisely coordinate virulence factor expression as changes in metal abundance can be associated with important bacterial life cycle events such as host entry or the arrival of recruited immune cells, and *H. pylori* is no exception. It is no surprise that several genes are regulated by more than one metal condition and concomitant regulators (Table 1). While two major regulators including Fur and NikR participate in *H. pylori* metal response and have been characterized in some depth, it is very likely that additional, as yet unknown, regulators exist and additional roles for the two major regulators have yet to be defined.

## Author contributions

KH and JG conceived, designed, wrote, reviewed, and edited this manuscript for critical content. Both authors approve this manuscript for integrity and accuracy.

### Conflict of interest statement

The authors declare that the research was conducted in the absence of any commercial or financial relationships that could be construed as a potential conflict of interest.
